# Increasing daily step count in mild cognitive impairment: a 12-month randomised controlled trial

**DOI:** 10.3389/fnagi.2026.1958222

**Published:** 2026-09-23

**Authors:** Małgorzata Jamka, Aleksandra Makarewicz-Bukowska, Mikołaj A. Pawlak, Joanna Popek, Edyta Mądry, Patrycja Krzyżanowska-Jankowska, Katarzyna Karmelita-Katulska, Anna Miśkiewicz-Chotnicka, Szymon Kurek, Radosław Trepanowski, Joanna Bajerska, Aleksandra Lisowska-Walkowiak, Ewa Wysocka, Marcin Żarowski, Jan Krzysztof Nowak, Karl-Heinz Herzig, Jarosław Walkowiak

**Affiliations:** 1 Poznan University of Medical Sciences, Department of Pediatric Gastroenterology and Metabolic Diseases, Poznań, Poland; 2 Poznan University of Medical Sciences, Department of Neurology, Poznań, Poland; 3 Department of Human Genetics, Radboud University Medical Center, Nijmegen, Netherlands; 4 Poznan University of Medical Sciences, Department of Physiology, Poznań, Poland; 5 Poznan University of Medical Sciences, Department of General Radiology and Neuroradiology, Poznań, Poland; 6 Faculty of Psychology and Cognitive Science, Adam Mickiewicz University, Poznań, Poland; 7 Department of Human Nutrition and Dietetics, Poznan University of Life Sciences, Poznań, Poland; 8 Poznan University of Medical Sciences, Department of Pediatric Diabetes, Auxology and Obesity, Poznań, Poland; 9 Poznan University of Medical Sciences, Department of Laboratory Diagnostics, Poznań, Poland; 10 Poznan University of Medical Sciences, Department of Developmental Neurology, Poznań, Poland; 11 Research Unit of Biomedicine and Internal Medicine, Faculty of Medicine, University of Oulu, Oulu, Finland; 12 Biocenter Oulu, University of Oulu, Oulu, Finland; 13 Medical Research Center, Oulu University Hospital, Oulu, Finland

**Keywords:** cognitive assessment, cognitive decline, neuroimaging, physical activity, walking, wearable technology

## Abstract

**Introduction:**

Despite numerous studies confirming the beneficial effects of physical activity on cognitive function, there is a lack of randomised controlled trials involving long-term, continuous monitoring of physical activity that assess the effect of step-based intervention on cognition-related outcomes. Therefore, this study evaluated whether increasing daily step count improves cognitive performance and brain health markers in individuals with mild cognitive impairment (MCI).

**Methods:**

Caucasian, community-dwelling participants aged 50–70 years with MCI and smartphone ownership were recruited for this randomised, controlled, parallel-group study, and assigned to intervention or control groups. The intervention group aimed to achieve ≥10,000 steps daily, with no individualised or baseline-adjusted activity targets, whereas controls maintained usual activity. Physical activity was monitored by wrist-worn trackers over 12 months. Cognitive function was measured using the Montreal Cognitive Assessment (MoCA) and the Cambridge Neuropsychological Test Automated Battery. Brain volume, cardiometabolic, and neurodegeneration biomarkers were assessed.

**Results:**

198 participants were recruited between July 2021 and September 2022 and assigned to the intervention (*n* = 98) or control (*n* = 100) group. 188 participants completed the study. Significant differences between groups were found in daily step count (median difference [95% confidence interval]: 1,728 (1,207–2,271), *p* < 0.0001), the MoCA (1 [0–2], *p* = 0.0043), spatial working memory errors in the six-box condition (−1 [−2–0], *p* = 0.0049), waist (−1 [−3–0] cm, *p* = 0.025), and hip circumferences (−1 [−2–0] cm, *p* = 0.047). No differences were observed in body composition, biochemical parameters, or brain volume parameters.

**Discussion:**

In adults with MCI and moderate baseline activity, a small increase in daily step count over 12 months led to modest changes in MoCA scores, while evidence from the broader cognitive battery was limited and largely exploratory. No effect on brain volume, peripheral neurodegeneration markers, body composition, or metabolism was noted (Trial registry: https://drks.de; identifier: DRKS00020943).

## 1 Introduction

Mild cognitive impairment (MCI) occurs both in normal ageing and as a disease stage preceding dementia, and it can affect one or more cognitive domains (Petersen et al., 2014). A recent meta-analysis estimated the global prevalence of MCI in individuals aged 50 years or over to be 19.7%, with a recent increase attributed to the coronavirus pandemic (Song et al., 2023). Factors associated with the prevalence of MCI include low education levels, living alone, alcohol consumption, obesity, diabetes, hypertension, brain injury, hearing loss, pollution, and depression (Livingston et al., 2024). Because there are currently no disease-modifying pharmacological treatments for MCI caused by degenerative diseases, modifying lifestyle-related factors may help prevent or delay the onset of MCI or slow its progression to dementia.

Physical activity is currently considered one of the key factors in the prevention of cognitive decline (Veronese et al., 2023). Among different types of physical activity, walking is considered one of the most accessible and preventive forms of exercise for the ageing population and has a positive effect on general health (Ding et al., 2025). Randomised controlled trials have also suggested that walking intervention may be associated with better white matter health (Mendez Colmenares et al., 2021) and increased hippocampal volume, alongside increased concentrations of brain-derived neurotrophic factor (Erickson et al., 2011). Yau et al. (2025) demonstrated in their longitudinal study that a higher number of daily steps is associated with slower cognitive decline in older adults with an elevated baseline amyloid burden. A higher number of daily steps was associated with slower temporal tau accumulation. This association also reached a plateau at a moderate level of physical activity (5,001–7,500 steps per day).

Despite numerous studies confirming the beneficial effects of physical activity on cognitive function, there is a lack of randomised controlled trials involving long-term, continuous monitoring of physical activity that assess the effect of step-based intervention on cognition-related outcomes. Therefore, this trial was designed to evaluate the effects of increased step-based physical activity, as assessed by a wrist-worn tracker, on cognitive function, brain volume, biochemical markers, anthropometric and body composition parameters in individuals with MCI. In the current trial, we hypothesised that increased activity would 1. Alleviate cognitive decline, 2. Preserve brain volume and ventricle size, 3. Maintain healthy biomarker levels, and 4. Improve anthropometric parameters and body composition.

## 2 Materials and methods

### 2.1 Study design and setting

This was a single-centre, single-blind, randomised, controlled, parallel-group Physical Activity (PA) PROTECT trial in which participants and intervention-delivery researchers were aware of the group allocation, whereas outcome assessors and data analysts were blinded to allocation. The study was conducted at the Poznan University of Medical Sciences, Poznań, Poland, from July 2021 to October 2023. The study protocol was approved by the Bioethics Committee of Poznan University of Medical Sciences (reference no. 47/20) and was conducted in accordance with the Declaration of Helsinki. Participants and the public were not involved in the design, conduct, or reporting of this trial. The study protocol was prospectively registered in the German Clinical Trials Register database (registration no. DRKS00020943, date of registration: 9 March 2020). The full version of the study protocol is available in the article by Jamka et al. (2021). The manuscript was prepared according to the Consolidated Standards of Reporting Trials statement (see Supplementary Table 1, Hopewell et al., 2025).

### 2.2 Participants

Individuals with MCI were recruited from medical centres and clinics across the Greater Poland region, Poland, in collaboration with clinic directors and physicians, as well as through newsletters, websites, posters, and email invitations sent to companies, offices, and institutions to reach their employees. The participants were provided with study information, including descriptions of the study purpose, potential benefits, and possible risks. They were informed that their participation was entirely voluntary and that they had the right to decline or withdraw from the study at any time without providing a reason. All participants gave written informed consent at the time of enrolment.

Before enrolment, potential participants attended a screening visit, during which physicians verified eligibility based on the study criteria. Cognitive function was assessed using the Montreal Cognitive Assessment (MoCA) test, and depressive symptoms were evaluated using the Hamilton Depression Rating Scale (HAM-D). Participants’ physical activity was monitored for at least one week before enrolment using the Fitbit Inspire HR tracker (Fitbit LLC, San Francisco, CA, USA). Baseline physical activity was measured to verify compliance with the eligibility criteria and to compare changes in activity over time (the pre-intervention and intervention periods). Sex was self-reported by participants, and all participants were Caucasian.

Eligible participants were community-dwelling adults aged 50–70 years with MCI, identified based on their MoCA test score of 19–26 points, who owned a smartphone required for physical activity monitoring.

Exclusion criteria were as follows:

- Physical activity: high baseline physical activity, defined as >150 min per week of moderate activity, >75 min of vigorous activity, or an equivalent combination (World Health Organization, 2020), and accumulated >10,000 steps/day. Both criteria had to be met for a participant to be excluded from the study. Physical activity was assessed over seven consecutive days using a Fitbit Inspire HR tracker (Fitbit, Inc., San Francisco, CA, USA), which provided measures of daily step count and minutes of moderate- and vigorous-intensity activity.
- Conditions: depression with HAM-D scores >13, use of cognitive-enhancing or psychotropic medications, alcohol dependence (>15 units per week), diagnosed psychiatric conditions, Parkinson’s disease, Alzheimer’s disease, dementia, anaemia, diabetes for ten or more years, chronic kidney or liver disease, a history cancer, including chemotherapy or radiotherapy within the last five years, a history of stroke, recent seizures (within the past two years), head trauma involving loss of consciousness or immediate confusion, unmanaged hypothyroidism, any chronic illness restricting cardiovascular or respiratory training/testing.
- Magnetic resonance imaging (MRI) contraindications: presence of a pacemaker, neurostimulator, or other metal implants, claustrophobia.
- Other limitations: blindness, deafness, language barriers, or any other condition that could hinder participation in or cooperation with the study protocol.

### 2.3 Randomisation and blinding

Participants were randomly assigned to the intervention or control group using a randomisation list generated by an independent researcher with computer software (RRApp Robust Randomisation App, Icahn School of Medicine at Mount Sinai, New York, NY, USA). Random block sizes of six were used, with stratification by sex and prevalence of diabetes. The allocation sequence was concealed from the researchers responsible for enrolment and assessment.

### 2.4 Intervention and control conditions

Participants were randomly assigned in a 1:1 ratio to the intervention or control group. The control group was instructed to maintain their usual daily activity levels throughout the 12-month intervention period, whereas the intervention group was encouraged and guided by the mobile application, study team members, and psychologists to increase their physical activity to at least 10,000 steps per day. Participants were instructed to achieve this goal as soon as possible. Individual goals to increase or progress activity were not established. This goal was also not modified based on participants’ baseline activity levels. No advice about walking intensity was provided. All participants were instructed to maintain their current diet. No interim analyses were performed during the one-year intervention. Study participants were instructed to report all adverse events related to the intervention to the research team.

Data collected from the Fitbit tracker were used to assess data quality and adherence to the study intervention. This information was gathered weekly through an application programming interface (API) connection. If a participant did not meet the physical activity goal, if physical activity data were unavailable, or if the tracker was not worn, study team members contacted participants via phone calls, messages, or emails to remind them of their activity goals and encourage adherence to the study protocol. Telephone contact was the primary form of contact. However, if contacting the participant this way was difficult or the participants specifically requested it, they were contacted via text message. If there was no response to the telephone or text message contact, an email was sent to the participant reminding them to meet their activity goals. In addition, weekly emails containing information on average physical activity levels were sent to all participants. Participants could also monitor their own activity using the tracker or the mobile phone app. Participants were not excluded from the study if they did not meet the physical activity recommendations, but only if they were unable to upload their activity data or wear the physical activity tracker for an extended period.

### 2.5 Outcomes

The primary outcomes were changes in cognitive functions assessed by the MoCA test.

The secondary outcomes included changes in:

- The Cambridge Neuropsychological Test Automated Battery (CANTAB): Delayed Matching to Sample (DMS), Motor Screening Task (MOT), Paired Associates Learning (PAL), Pattern Recognition Memory (PRM), Reaction Time (RTI), Rapid Visual Information Processing (RVP), and Spatial Working Memory (SWM);
- Brain structure volume parameters: cortex, total and subcortical grey matter, lateral ventricle, and hippocampal volumes;
- Biochemical parameters: glucose, insulin, lipid profile, brain-derived neurotrophic factor (BDNF), β-amyloids 1–40 and 1–42, β-amyloid 1–42/1–40 ratio, C-reactive protein (CRP), interleukins (IL) 6 and 1R1, and tumour necrosis factor α (TNF-α);
- Blood pressure: systolic (SBP) and diastolic blood pressure (DBP), and heart rate (HR);
- Anthropometric parameters: body weight, body mass index (BMI), waist and hip circumferences, and waist-to-hip ratio (WHR);
- Body composition: fat mass (FM), visceral adipose tissue (VAT), lean mass index (LMI), and appendicular lean mass index (ALMI).

All outcomes were assessed pre- and post-intervention.

#### 2.5.1 Physical activity assessment

Physical activity was measured for at least 7 days before enrolment and continuously throughout the 12-month intervention period using the Fitbit Inspire HR tracker (Fitbit, Inc., San Francisco, CA, USA), a wrist-worn physical activity tracker that syncs with the Fitbit app, allowing users to monitor their activity. Participants were instructed to update the application whenever a new version became available. Firmware updates were managed according to the manufacturer’s standard procedures. The tracker was worn on the non-dominant wrist, and the Fitbit app was installed on the participants’ smartphones, with the participants instructed to wear the device throughout the day and synchronise their activity data before going to bed via the app daily. Individual user accounts were created for each participant on the Fitbit platform. Physical activity data were collected via the Fitbit API, including total energy expenditure, energy expenditure from physical activity, step count, distance travelled, sedentary time, and time spent in light, moderate, and vigorous activity. A valid monitoring day was defined as a day with at least 500 steps recorded (Mendoza et al., 2017). Because reliable non-wear information was not available, reliably distinguishing between true low-activity days and non-wear days was not possible.

#### 2.5.2 Cognitive assessment

MCI was identified using the MoCA scale (version 8.1 Polish [Poland]), with a score of 19–26 indicating MCI, 27–30 indicating normal cognitive function, and ≤19 suggesting dementia (Nasreddine et al., 2005). The assessments were conducted during the screening visit and post-intervention. The same version of the MoCA scale was administered pre- and post-intervention. All assessors successfully completed a one-hour online training and certification to administer and score the MoCA organised by MoCA Test Inc. (Québec, Canada). The assessment was carried out in a separate room, in which only the assessor and participant were present. Efforts were made to ensure that both assessments (pre- and post-intervention) were performed at a similar time of day.

The following outcomes were assessed in each CANTAB test:

- DMS: percent correct (simultaneous, 0-s delay, 4-s delay, 12-s delay);
- MOT: mean latency;
- PAL: total errors (adjusted), first-attempt memory score;
- PRM: percent correct immediate, percent correct delayed;
- RTI: median five-choice reaction time, median five-choice movement time;
- RVP: A’, false alarm probability, median response latency;
- SWM: strategy, between errors (4, 6, and 8 boxes).

The tests were run in the following order: MOT, RTI, PAL, PRM, SWM, RVP, and DMS. The tests used in the study has been validated in individuals with MCI (Campos-Magdaleno et al., 2021; Juncos-Rabadán et al., 2014; Nabizadeh et al., 2026; Sabahi et al., 2022). The assessment took place in a quiet, private room. The battery uses a Polish-recorded voice for instructions. The measurement was administered by a trained psychologist, who explained the assessment’s aim and procedures to the participant and observed the session without interfering with the participant’s responses. Participants’ responses were recorded using a 6th-generation iPad (Apple Inc., Cupertino, CA, USA). Programming updates were performed automatically according to the manufacturer’s recommendations. The average duration of the tests was approximately 60 min. For details, see the Supplementary material.

#### 2.5.3 MRI acquisition and processing

Brain images were acquired at the University Clinical Hospital (Przybyszewskiego Str. 49, 60–355 Poznań, Poland), using a Magnetom Skyra 3.0 T scanner (Siemens, Erlangen, Germany) equipped with syngo MR E11 software and a standard head coil. The following sequences were used: 3D T1-weighted magnetisation prepared rapid acquisition gradient echo acquisition: repetition time = 2,300 ms, echo time = 2.26 ms, inversion time = 900 ms, flip angle = 8^°^, field of view = 256 × 256, slice thickness = 1 mm, and 3D fluid-attenuated inversion recovery sampling perfection with application-optimised contrasts using different flip angle evolutions acquisition: repetition time = 5,000 ms, echo time = 338 ms, inversion time = 1,600 ms, flip angle = 120^°^, field of view = 256 × 256, and slice thickness = 1 mm. DICOM to NIFTI format conversion was performed using the dcm2niix software (Li et al., 2016). Six degrees of freedom registration of fluid-attenuated inversion recovery to T1 images was performed using the FreeSurfer 7.4 mri_coreg tool and further segmented using Sequence Adaptive Multimodal SEGmentation (Cerri et al., 2021) to extract white matter lesion volume, the volume of the cerebral cortex, and other cerebral structures. The data were converted to CSV format and imported into the common database, with analysts blinded to the randomisation results and physical performance during the trial. The following volumetric parameters (in mm^3^) were analysed: cortex volume, total grey matter volume, subcortical grey matter volume, total lateral ventricle volume, and total hippocampus volume. Longitudinal assessment of volume change was computed based on a more precise pipeline for multipoint evaluation based on a common spatial transformation (Reuter et al., 2012).

#### 2.5.4 Blood sampling and biochemical analyses

Blood samples were collected in the morning after a 12-h fast from the antecubital vein via standard venipuncture performed by registered nurses to measure biochemical parameters. The following parameters were measured in serum: insulin, lipid profile, CRP, BDNF, TNFα, IL1R1, and IL-6, while glucose, amyloid β-40 and β-42, and tau protein were assessed in plasma. Glucose, insulin, lipid profile (total cholesterol [TC], low-density lipoprotein cholesterol [LDL-C], high-density lipoprotein cholesterol [HDL-C], non-HDL-C [TC – HDL-C], triglycerides [TG]), and CRP were analysed in a commercial laboratory (Diagnostyka S.A., Poznań, Poland). Glucose levels were evaluated using the enzymatic method, insulin concentrations were measured using the chemiluminescent microparticle immunoassay method and the lipid profile was analysed using a calorimetric method. CRP levels were measured using a high-sensitivity immunoturbidimetry method. The remaining parameters were assessed by the enzyme-linked immunosorbent assay (ELISA) method in the laboratory of the Department of Pediatric Gastroenterology and Metabolic Diseases, Poznan University of Medical Sciences, Poznań, Poland. The following kits were used: Human IL1R1 ELISA Kit, LSBio; TNF-alfa Human ELISA Kit, DRG International Inc.; Human Tau proteins ELISA kit, Cusabio; Human Free BDNF Quantikine ELISA Kit, R&D Systems; Human ELISA Kit high-sensitivity IL-6 HS, R&D Systems; and Amyloid-beta (1–40) ELISA, IBL; Amyloid-beta (1–42) ELISA, IBL. The tests were performed in accordance with the principles of Good Clinical Laboratory Practice.

#### 2.5.5 Blood pressure, anthropometry, and body composition

SBP and DBP were measured on the left arm after a few minutes of rest in the sitting position, following the guidelines of the European Society of Hypertension (Kreutz et al., 2024). HR was also assessed. All measurements were taken using an electronic blood pressure monitor (Omron M2, Kyoto, Japan).

Weight, height, hip, and waist circumferences were assessed according to standardised procedures during the pre- and post-intervention periods to calculate BMI and WHR. The measurements were taken with participants wearing light clothing and barefoot. A Radwag WPT 100/200 OW (Radom, Poland) scale with a stadiometer was used to measure body weight and height, and a Seca 201 tape (Hamburg, Germany) was used to measure waist and hip circumferences. Body weight was measured to the nearest 0.1 kg, while body height, waist, and hip circumferences were measured to the nearest 0.1 cm. Two measurements were taken, and their mean was used for analysis. Waist circumference was assessed midway between the lowest rib and the uppermost border of the iliac crest. Hip circumference was measured at the level of the largest circumference of the buttocks (World Health Organization, 2011).

Body composition was assessed in the morning using dual-energy X-ray absorptiometry with the Hologic Discovery QDR analyser, model Wi (Bedford, MA, USA). FM (g) and percentage of FM (%FM) were measured. Lean indices included LMI and ALMI. In addition, VAT was estimated. Daily quality control procedures were performed before scanning participants to ensure the proper functioning of the system and software. Measurements were performed in accordance with the manufacturer’s guidelines. All operators performing scans were appropriately trained and certified in patient radiation protection. Participants were scanned in the supine position while wearing light clothing and were asked to remove all metal objects before the examination. Participants were instructed to avoid intense physical activity immediately before the examination, and measurements were performed at least 3 h after the last meal.

#### 2.5.6 Other parameters

The 17-item HAM-D scale was administered by a physician at the screening visit to evaluate the presence of depressive symptoms, with each item scored from 0 to 2 or 0 to 4 resulting in a possible total score ranging from 0 to 52. The following cut-off points were applied: <7 points, no depression; 8–13 points, mild depression; 14–18 points, moderate depression; 18–22 points, severe depression; and ≥23 points, very severe depression (Hamilton, 1960).

The participants completed a survey at baseline to collect demographic information, including their place of residence, education, employment status, financial situation, smoking habits, alcohol consumption, and medication use.

Dietary habits were evaluated before and during the intervention by analysing three-day food diaries that included two weekdays and one weekend day. Participants were instructed to provide detailed descriptions and quantities of all consumed foods. Dietary nutritional value was analysed using Aliant software (Anmarsoft, Gdańsk, Poland).

### 2.6 Statistical analysis

The minimum required sample size was determined using G*Power 3.1 software (Heinrich Heine University Düsseldorf, Düsseldorf, Germany) based on findings from a physical activity intervention study by Zhu et al. (2017). The required sample size was 79 participants per group based on the following assumptions: 1. a type-I error probability at *α* = 0.05, 2. a type-II error probability at *β* = 0.2, 3. a mean difference of 0.62 standard deviations (SDs), and 4. an expected SD of 85% of the mean. A total of 198 participants were recruited, considering a 20% dropout rate.

The primary endpoint was the change in MoCA score over the 12-month intervention. Secondary efficacy endpoints included changes in CANTAB tests results, brain volume, blood pressure, biochemical markers, anthropometric parameters, and body composition. The within-group differences were calculated for each group as post-intervention minus pre-intervention values. Subsequently, the between-group difference was calculated as the difference between the within-group changes (intervention minus control).

Statistical analyses were performed using Statistica 13.0 (TIBCO Software Inc., Palo Alto, CA, USA) and Stata/SE 19.5 (StataCorp, College Station, TX, USA), with a two-sided *p* value of <0.05 indicating statistical significance. The Shapiro–Wilk test was employed to assess variable normality. Due to the non-parametric distribution of the data, descriptive statistics for the study population characteristics are presented as medians and interquartile ranges (IQR; Q1–Q3) or as frequencies and percentages. The median difference with 95% confidence intervals (95%CI) was also calculated using a percentile-based method. The Mann–Whitney U test was used for unpaired comparisons between two groups, while the Wilcoxon test assessed intragroup changes from baseline, and the χ^2^ test was used to assess relationships between categorical variables. Spearman’s correlation coefficient was calculated to investigate the association between sedentary behaviour and moderate-to-vigorous physical activity separately for each group. No imputation of missing data was performed. Subgroup analyses and adjusted analyses were not performed. No adjustment for multiple comparisons was made. Therefore, the analyses of secondary outcomes should be considered exploratory. Accordingly, the analysis of baseline characteristics followed an intention-to-treat principle, whereas the analysis of changes from baseline was conducted using the participants with available pre- and post-intervention data and should be considered a complete-case analysis.

## 3 Results

Caucasian, community-dwelling participants aged 50–70 years with MCI and who owned a smartphone were recruited from July 2021 to September 2022 and attended enrolment, pre-intervention and post-intervention visits. Initially, a follow-up visit was planned in the sixth month of the intervention, but it was cancelled because of the coronavirus pandemic and restrictions on access to the hospital where the study centre was located. The cancellation of the six-month visit was a protocol deviation but did not result in any additional interruptions to or modifications of the intervention. Participant flow is presented in Figure 1, with 968 participants assessed for eligibility, of whom 770 were excluded (632 did not meet the inclusion criteria, 18 declined to participate, and 120 were excluded for other reasons). Ultimately, 198 Caucasian participants were included in the study and randomised to the intervention (*n* = 98) and control (*n* = 100) groups. During the intervention, five participants from the intervention group and four from the control group withdrew. In the intervention group, one participant withdrew without providing a reason, while four withdrew due to health issues unrelated to the intervention. In the control group, all individuals withdrew due to health issues unrelated to the study. In the intervention group, participants withdrew from the study at weeks 6, 8, 19, 28, and 51, while in the control group, participants withdrew at each of weeks 6, 11, 13, and 18. One participant from the control group was lost in follow-up; thus, 93 participants from the intervention group and 95 from the control group completed the study. The baseline demographic and clinical characteristics are presented in Tables 1, 2.

**Figure 1:**
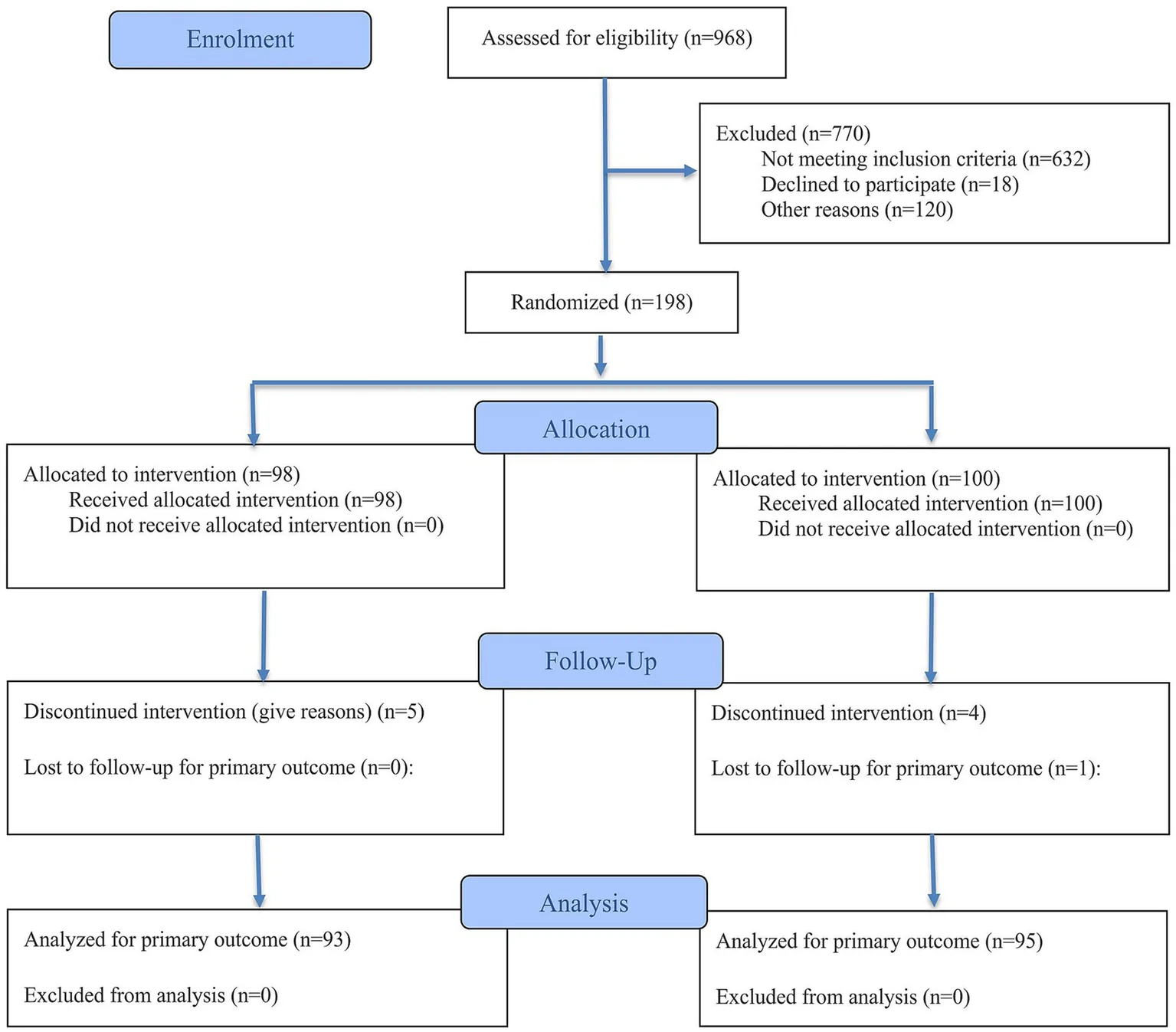
CONSORT flow chart (Hopewell et al., 2025). Adapted under the terms of the Creative Commons Attribution 4.0 International License (CC BY 4.0). The diagram presents the progress through the phases of a randomised trial of two groups, including enrolment, intervention allocation, follow-up, and data analysis.

**Table 1:** Baseline clinical characteristics of participants by study group.

Variables	Intervention group (*n* = 98), median(Q1–Q3)	Control group (*n* = 100), median(Q1–Q3)	*p*
Anthropometric parameters and body composition			
Age [years]	59 (53–63)	59 (54–63)	0.50
Weight [kg]	73.2 (65.8–86.0)	75.0 (64.5–82.5)	0.83
Height [m]	1.64 (1.59–1.72)	1.64 (1.60–1.70)	0.96
BMI [kg/m^2^]	27.0 (25.2–28.9)	27.4 (24.5–30.4)	0.80
Waist circumference [cm]	92.0 (84.0–101.0)	93.0 (84.5–99.5)	0.83
Hip circumference [cm]	105.0 (100.0–110.0)	105.0 (100.0–110.0)	0.96
WHR	0.88 (0.81–0.93)	0.89 (0.82–0.94)	0.70
Total body			
FM [g]	26,545 (22,230 – 30,355)	25,968 (21,243 – 32,633)	0.91
FM [%]	36.8 (32.1–41.4)	38.1 (31.7–41.5)	0.72
VAT			
Mass [g]	660 (451–827)	631 (444–814)	0.62
Volume [cm^3^]	714 (487–894)	683 (480–880)	0.62
Area [cm^2^]	137.0 (93.4–171.0)	131.0 (92.1–169.0)	0.63
LMI [kg/m^2^]	15.6 (14.6–17.5)	15.8 (14.2–17.9)	0.88
ALMI [kg/m^2^]	6.63 (6.17–7.61)	6.66 (5.92–7.74)	0.71
Blood pressure and biochemical parameters^a^			
Blood pressure			
SBP [mmHg]	127 (117–140)	128 (118–140)	0.83
DBP [mmHg]	80 (72–86)	80 (73–87)	0.84
HR [bpm]	73 (67–80)	71 (65–80)	0.22
Glucose and insulin homeostasis			
Glucose [mg/dl]	93 (89–100)	95 (89–103)	0.25
Insulin [μIU/ml]	6.2 (4.5–8.0)	6.4 (4.6–9.6)	0.38
Lipid profile			
TC [mg/dl]	216 (190–236)	221 (194–249)	0.24
HDL-C [mg/dl]	58 (47–65)	56 (48–66)	0.76
non-HDL-C [mg/dl]	159 (128–179)	160 (136–186)	0.52
LDL-C [mg/dl]	133 (109–156)	139 (116–164)	0.40
TG [mg/dl]	105 (73–146)	99 (76–136)	0.77
Inflammatory parameters			
CRP [mg/l]	1.5 (0.7–3.1)	1.0 (0.6–1.7)	0.017
IL-6 [pg/ml]	1.5 (1.1–2.2)	1.3 (0.9–1.7)	0.025
IL1R1 [pg/ml]	233.2 (132.1–437.6)	205.4 (132.9–376.4)	0.35
TNF-α [pg/ml]	7.9 (6.5–9.5)	7.9 (6.6–9.1)	0.90
Peripheral neurodegeneration parameters			
BDNF [ng/ml]	31.1 (26.8–35.7)	32.2 (28.3–36.1)	0.29
Tau protein [pg/ml]	35.6 (19.6–51.5)	34.8 (21.8–47.8)	0.79
β-amyloid 1–40 [pg/ml]	168.8 (139.7–202.8)	173.0 (133.0–220.2)	0.38
β-amyloid 1–42 [pg/ml]	4.9 (3.3–7.8)	5.3 (3.3–8.0)	0.97
β-amyloid 1–42/1–40	0.03 (0.02–0.05)	0.03 (0.02–0.04)	0.67
Physical activity parameters			
Calories [kcal/day]			
Total	2,130 (1,880–2,504)	2,184 (1,912–2,502)	0.61
Fom activity[kcal/day]	993 (887–1,223)	1,101 (910–1,248)	0.23
Steps/day	8,987 (7,244–10,571)	9,529 (8,223–10,511)	0.26
Distance [km]	6.1 (5.1–7.3)	6.5 (5.6–7.2)	0.37
Physical activity [min/day]			
Light	277 (224–311)	285 (243–337)	0.12
Moderate	14 (7–20)	14 (8–20)	0.74
Vigorous	13 (7–23)	11 (7–22)	0.41
Sedentary behaviour [min/day]	689 (648–749)	676 (616–729)	0.14
Cognitive function tests			
MoCA score [points]	25 (23–26)	25 (23–26)	0.69
HAM-D-17 score [points]	2 (1–5)	3 (1–5)	0.66
DMS			
Percent correct, simultaneous	87 (80–95)	90 (85–95)	0.55
Percent correct, 0-s delay	100 (80–100)	100 (80–100)	0.26
Percent correct, 4-s delay	80 (60–100)	80 (80–100)	0.30
Percent correct, 12-s delay	80 (60–100)	80 (60–100)	0.36
Probability of error given error	0.0 (0.0–0.2)	0.0 (0.0–0.0)	0.50
MOT			
Mean latency [ms]	793.8 (730.4–932.6)	814.4 (729.9–976.1)	0.53
PAL			
Total errors, adjusted	19 (10–31)	21 (12–36)	0.24
First-attempt memory score	10 (8–12)	10 (7–13)	0.29
PRM			
Percent correct immediate	88.9 (77.8–94.4)	88.9 (77.8–94.4)	0.58
Percent correct delayed	83.3 (77.8–94.4)	83.3 (72.2–88.9)	0.065
RTI			
Median five-choice reaction time [ms]	405.7 (376.6–431.1)	407.2 (374.7–432.5)	0.80
Median five-choice movement time [ms]	307.5 (264.5–367.9)	297.8 (256.8–345.7)	0.097
RVP			
A’	0.8771 (0.8438–0.9120)^3^	0.8713 (0.8386–0.8999)	0.32
False alarm probability	0.0058 (0.0021–0.0109)	0.0071 (0.0025–0.0253)	0.047
Median response latency [ms]	501.8 (463.3–576.3)	537.8 (462.0–606.8)	0.19
SWM			
Strategy	10 (8–11)	10 (9–11)	0.36
Between errors, 4 boxes	1 (0–2)	1 (0–2)	0.81
Between errors, 6 boxes	6 (2–8)	5 (2–7)	0.16
Between errors, 8 boxes	12 (7–15)	14 (8–15)	0.14
Brain volume^b^			
Cortex volume [mm^3^]	448,837 (432,624 – 482,610)	449,963 (423,321 – 474,957)	0.41
Total grey matter volume [mm^3^]	609,670 (588,506 – 649,179)	610,969 (581,481 – 642,615)	0.64
Subcortical grey matter volume [mm^3^]	58,392 (55,280 – 62,279)	58,441 (55,637 – 62,120)	0.96
Total lateral ventricle [mm^3^]^c^	8,806 (6,273–12,207)	8,158 (6,563–11,723)	0.78
Total hippocampus [mm^3^]^c^	4,217 (3,991–4,542)	4,232 (3,916–4,588)	0.76

ALMI, appendicular lean mass index; BDNF, brain-derived neurotrophic factor; BMI, body mass index; CRP, C reactive protein; DBP, diastolic blood pressure; DMS, delayed match to sample; FM, fat mass; HAM-D, Hamilton Depression Rating Scale; HDL-C, high density lipoprotein cholesterol; HR, heart rate; IL, interleukin; LDL-C, low density lipoprotein cholesterol; LMI, lean mass index; MoCA, Montreal Cognitive Assessment Scale; MOT, motor screening task; non-HDL-C – total cholesterol minus high density lipoprotein cholesterol; PAL, paired associates learning; PRM, pattern recognition memory; Q1 – Q3, interquartile range; RTI, reaction time task; RVP, rapid visual information processing; SBP, systolic blood pressure; SWM, spatial working memory; TC, total cholesterol; TG, triglycerides; TNF-α, tumour necrosis factor α; VAT, visceral adipose tissue; WHR, waist-to-hip ratio.

a Control group *n* = 99 – one subject refused blood sampling.

b Intervention group *n* = 96 and Control group *n* = 98— in four subjects, the measurement was not performed because the subjects were unable to lie still during the analysis, had previously unrecognised claustrophobia, or had other contraindications to the examination that they either did not disclose or were unaware of at the time of study qualification.

c (Left+right)/2.

**Table 2:** Baseline sociodemographic characteristics of participants by study group.

Variables	Intervention group (*n* = 98), n (%)	Control group (*n* = 100), n (%)	*p*
Sex			
Women	72 (73.5)	77 (77.0)	0.62
Men	26 (26.5)	23 (23.0)	
Place of residence			
Village	23 (23.5)	20 (20.0)	0.72
City < 50,000 inhabitants	14 (14.3)	11 (11.0)	
City of 50,000–500,000 inhabitants	10 (10.2)	9 (9.0)	
City > 500,000 inhabitants	51 (52.0)	60 (60.0)	
Family situation			
Formal/informal relationship	72 (73.5)	71 (71.)	0.70
Single	26 (26.5)	29 (29.0)	
Education			
Primary	2 (2.0)	1 (1.0)	0.62
Vocational	3 (3.1)	3 (3.0)	
Secondary	21 (21.4)	29 (29.0)	
High	72 (73.5)	67 (67.0)	
Socio-occupational status			
Employed	79 (80.6)	76 (76.0)	0.53
Unemployed	1 (1.0)	3 (3.0)	
Pensioner	18 (18.4)	21 (21.0)	
Financial situation			
Very good	7 (7.1)	8 (8.0)	0.17
Good	72 (73.5)	60 (60.0)	
Mediocre	19 (19.4)	31 (31.0)	
Bad	0 (0.0)	1 (1.0)	
Smoking (currently)			
Yes	17 (17.5)	12 (12.1)	0.32
No	80 (82.5)	87 (87.9)	
Smoking (in the past)			
Yes	41 (42.3)	49 (49.5)	0.32
No	56 (57.7)	50 (50.5)	
Alcohol consumption			
Yes	78 (79.4)	77 (76.8)	0.77
No	20 (20.6)	23 (23.2)	
Type of alcohol consumed most often			
Beer	20 (28.6)	13 (17.1)	0.22
Wine	38 (54.3)	53 (69.7)	
Drinks	9 (12.9)	6 (7.9)	
Hard liquor	3 (4.3)	4 (5.3)	
Antihypertensive drugs			
Yes	28 (28.6)	36 (36.0)	0.29
No	70 (71.4)	64 (64.0)	
Hypolipidemic drugs			
Yes	13 (13.3)	18 (18.0)	0.44
No	85 (86.7)	82 (82.0)	
Hypoglycaemic drugs			
Yes	6 (6.1)	11 (11.0)	0.31
No	92 (93.9)	89 (89.0)	
Hypothyroidism drugs			
Yes	11 (11.2)	10 (10.0)	0.82
No	87 (88.8)	90 (90.0)	
Hormone replacement therapy			
Yes	2 (2.0)	0 (0.0)	1.000
No	96 (98.0)	100 (100.0)	

Adverse events were reported in 14 participants in the intervention group and 15 participants in the control group. The reported adverse events included: orthopaedic problems (6 [42.9%] vs. 6 [40.0%]), nervous system disorders (1 [7.1%] vs. 3 [20.0%]), cancer (2 [14.3%] vs. 1 [6.7%]), skin lesions (2 [14.3%] vs. 0 [0.0%]), infectious diseases (2 [14.3%] vs. 0 [0.0%]) and other (1 [7.1%] vs. 1 [6.7%]) in the intervention and control groups, respectively. No serious adverse events related to the study intervention were reported during the intervention period.

Differences in physical activity levels between groups during the intervention period are presented in Table 3. Significant between-group differences were observed in step count (*p* < 0.0001), distance (*p* < 0.0001), total calorie expenditure (*p* = 0.017), activity-related calorie expenditure (*p* = 0.0017), and minutes of vigorous physical activity (*p* < 0.0001). Among individuals who completed the study, 87.4% from the control group and 98.9% from the intervention group (*p* = 0.0025) successfully implemented physical activity guidelines to perform at least 150 min per week of moderate activity, 75 min of vigorous activity, or an equivalent combination of both (World Health Organization, 2020). The frequency of contact with participants during the intervention period due to missing physical activity data or failure to meet physical activity goals did not differ between the groups (control group vs. intervention group: median [Q1 – Q3]: 15 [3–28] vs. 19 [5–39], *p* = 0.12). Among participants who completed the study, the median percentage of days excluded from the analysis due to a step count ≤ 500 did not differ significantly between groups (*p* = 0.12): 0.3 (0.0–1.9)% in the intervention group and 0.8 (0.0–3.8)% in the control group. 60.2% of participants in the intervention group and 31.6% in the control group achieved a mean of ≥10,000 steps per day (*p* < 0.0001). Participants in the intervention group met the target on a median of 60.6 (Q1 – Q3: 38.1–80.0)% of valid days. Adherence changed over the course of the intervention, with a decrease observed midway through the period. Detailed data on changes in physical activity over the intervention period are presented in Supplementary Figures 1–5. Moreover, the relationship between average sedentary behaviuor and moderate-to-vigorous physical activity over the 12-month observation period is presented in Supplementary Figures 6A,B.

**Table 3:** Comparison of physical activity levels between groups during the 12-month intervention.

Variables	Intervention group (*n* = 93), median (Q1–Q3)	Control group (*n* = 95), median (Q1 – Q3)	*p*
Calories [kcal/day]			
Total	2,203 (1,970–2,647)	2,139 (1,850–2,433)	0.017
From activity	1,143 (992–1,324)	1,033 (831–1,189)	0.0017
Steps/day	10,612 (8,959–12,443)	9,040 (7,241–10,431)	<0.0001
Distance [km]	7.3 (6.3–8.4)	6.2 (5.0–7.0)	<0.0001
Physical activity [min/day]			
Light	281 (243–317)	268 (230–317)	0.33
Moderate	18 (13–23)	15 (11–23)	0.10
Vigorous	22 (15–30)	14 (10–22)	<0.0001
Sedentary behaviour [min/day]	669 (618–730)	692 (644–757)	0.069

Q1–Q3, interquartile range.

Between-group differences in primary and secondary outcomes are presented in Tables 4–7. As expected, the groups differed significantly in changes in physical activity levels: (*p* < 0.05 for all; Table 4). The intervention group increased their activity, including daily step counts, whereas the control group showed a slight decrease over the intervention period (median difference [95%CI]: 1,728 [1,207 to 2,271], *p* < 0.0001). MoCA test scores changed in the intervention group compared with unchanged scores in the control group (median difference [95%CI]: 1 [0 to 2], *p* = 0.0043, Table 5). SWM test outcomes also differed, specifically in the number of times participants revisited boxes where tokens had previously been found (calculated across trials with six boxes only, median difference [95%CI]: −1 [−2 to 0], *p* = 0.0049, Table 5). However, the clinical relevance of this change remains uncertain. The differences between groups in MRI results did not reach statistical significance (Table 6).

**Table 4:** Comparison of changes (Δ; during intervention minus pre-intervention) in objectively measured (Fitbit Inspire HR) physical activity between the intervention and control groups.

Variables	Intervention group (*n* = 93), median (Q1–Q3)	Control group (*n* = 95), median (Q1–Q3)	Median differences (95%CI)	*p*
Calories [kcal/day]				
Total	66 (−26–168)	−46 (−108–32)	111 (69–157)	<0.0001
From activity	112 (−20–222)	−19 (−108–82)	127 (76–177)	<0.0001
Steps/day	1,474 (207–3,313)	−176 (−1,100–846)	1,728 (1,207–2,271)	<0.0001
Distance [km]	1.0 (0.1–2.2)	0.0 (−0.8–0.6)	1.2 (0.8–1.6)	<0.0001
Physical activity [min/day]				
Light	5 (−14–33)	−11 (−31–9)	19 (9–30)	0.0005
Moderate	5 (−2–10)	1 (−3–6)	3 (0–5)	0.029
Vigorous	7 (1–13)	2 (−3–7)	5 (3–8)	0.0001
Sedentary behaviour [min/day]	−18 (−66–36)	19 (−17–63)	−39 (−60– −17)	0.0004

CI, confidence interval; Q1–Q3, interquartile range.

**Table 5:** Comparison of changes (Δ; post-intervention minus pre-intervention) in cognitive test results between the intervention and control groups.

Variables	Intervention group (*n* = 92)^a^, median (Q1–Q3)	Control group (*n* = 95), median (Q1–Q3)	Median differences (95%CI)	*p*
MoCA score [points]	1 (0–2)^b^	0 (−2–2)	1 (0–2)	0.0043
DMS				
Percent correct, simultaneous	0 (0–0)	0 (0–0)	0 (0–0)	0.98
Percent correct, 0-s delay	0 (−20–20)	0 (−20–0)	0 (0–0)	0.32
Percent correct, 4-s delay	0 (0–20)	0 (0–20)	0 (0–0)	0.25
Percent correct, 12-s delay	0 (−20–20)	0 (−20–20)	0 (0–0)	0.73
Probability of error given error	0.0 (0.0–0.0)	0.0 (0.0–0.2)	0 (0–0)	0.18
MOT				
Mean latency [ms]	−14.6 (−122.9–81.6)	−18.8 (−143.3–52.3)	18.6 (−31.0–65.9)	0.47
PAL				
Total errors, adjusted	−3 (−12–2)	−4 (−12–2)	1 (−2–4)	0.58
First-attempt memory score	2 (−1–3)	1 (−1–3)	0 (−1–1)	0.52
PRM				
Percent correct immediate	0.0 (−5.6–11.1)	0.0 (−5.6–11.1)	0.0 (0.0–5.6)	0.50
Percent correct delayed	0.0 (−11.1–11.1)	0.0 (−5.6–11.1)	0.0 (−5.6–5.6)	0.61
RTI				
Median five-choice reaction time [ms]	0.5 (−20.0–18.0)	−5.0 (−28.0–15.5)	4.0 (−5.5–13.0)	0.43
Median five-choice movement time [ms]	−8.0 (−36.0–33.5)	13.0 (−23.5–41.5)	−16 (−33–1.5)	0.071
RVP				
A’	0.0196 (−0.0055–0.0464)	0.0094 (−0.0166–0.0441)	0.0091 (−0.0025–0.0212)	0.11
False alarm probability	0.0000 (−0.0051–0.0043)	−0.0020 (−0.0089–0.0020)	0.0021 (0.0000–0.0053)	0.081
Median response latency [ms]	−11.5 (−54.0–30.0)	−6.0 (−64.0–36.5)	−3.5 (−24.5–18.5)	0.77
SWM				
Strategy	−1 (−2–1)	0 (−2–1)	0 (−1–0)	0.085
Between errors, 4 boxes	0 (−2–0)	0 (−1–1)	0 (−1–0)	0.21
Between errors, 6 boxes	−1 (−4–0)	0 (−2–2)	−1 (−2–0)	0.0049
Between errors, 8 boxes	−2 (−5–3)	0 (−4–5)	−2 (−4–0)	0.061

CI, confidence interval; DMS, delayed match to sample; MoCA, Montreal Cognitive Assessment Scale; MOT, motor screening task; PAL, paired associates learning; PRM, pattern recognition memory; Q1 – Q3, interquartile range; RTI, reaction time task; RVP, rapid visual information processing; SWM, spatial working memory.

a One participant declined to complete the Cambridge Neuropsychological Test Automated Battery (CANTAB) examination at the end of the intervention.

b *n* = 93.

**Table 6:** Comparison of changes (Δ; post-intervention minus pre-intervention) in brain quantitative structural measures derived from magnetic resonance imaging between the intervention and control groups.

Variables	Intervention group (*n* = 93), median (Q1–Q3)	Control group (*n* = 94)^a^, median (Q1–Q3)	Median differences (95%CI)	*p*
Cortex volume [mm^3^]	−3,020 (−8,765–2,663)	−2,730 (−8,437–2,286)	−261 (−2,652–2,035)	0.85
Total grey matter volume [mm^3^]	−4,896 (−10,546–3,457)	−4,794 (−11,364–1,151)	871 (−1,769–3,456)	0.52
Subcortical grey matter volume [mm^3^]	−186 (−598–153)	−281 (−652–101)	82 (−94–252)	0.38
Total lateral ventricle [mm^3^]^b^	227 (6–501)	230 (−9–467)	−5 (−123–114)	0.93
Total hippocampus [mm^3^]^b^	−9 (−51–38)	−26 (−68–14)	19 (−2–39)	0.086

CI, confidence interval; Q1–Q3, interquartile range.

a One participant refused to undergo the examination at the end of the intervention.

b (Left+right)/2.

However, several differences in cognitive function and neuroimaging parameters were observed between pre- and post-intervention assessments in both groups. Cognitive function, assessed by the MoCA test, significantly improved in the intervention group (*p* < 0.0001). Additionally, significant changes between pre- and post-intervention values in both groups were noted for adjusted total errors (*p* < 0.001) and first-attempt memory scores (*p* < 0.01) in the PAL test and A’ in the RVP test (*p* < 0.05). Moreover, in the intervention group, the percentage correct after a 4-s delay in the DMS test (*p* = 0.021), the percentage correct immediately in the PRM test (*p* = 0.020), and all five SWM test parameters (*p* < 0.05) significantly differed between pre- and post-intervention values. In the control group, there were significant differences in scores on the MOT (*p* = 0.028), PAL (*p* < 0.0001), probability of a false alarm in the RVP test (*p* = 0.0046), and the median five-choice movement time in the RTI test (*p* = 0.044, Supplementary Table 2). Thus, in the intervention group, improvements were observed in ten parameters, whereas in the control group, only five parameters improved.

Importantly, cortex volume (*p* = 0.0008), total grey matter volume (*p* ≤ 0.0001), subcortical grey matter volume (*p* = 0.0003 and *p* < 0.0001, respectively), and total lateral ventricle volume (*p* < 0.0001) differed significantly between pre- and post-intervention values in both groups. Total hippocampus volume decreased only in the control group but not in the intervention group (*p* = 0.0004, Supplementary Table 3).

Between-group comparisons revealed significant differences in changes in waist (median difference [95%CI]: −1 [−3 to 0], *p* = 0.025) and hip (median difference [95%CI]: −1 [−2 to 0], *p* = 0.047) circumferences, with a greater reduction observed in the intervention group. In contrast, no between-group differences were detected in body composition (Table 7), blood pressure, or biochemical parameters (Table 8).

**Table 7:** Comparison of changes (Δ; post-intervention minus pre-intervention) in anthropometric parameters and body composition between the intervention and control groups.

Variables	Intervention group (*n* = 93), median (Q1–Q3)	Control group (*n* = 95), median (Q1–Q3)	Median differences (95%CI)	*p*
Weight [kg]	0.0 (−2.2–2.0)	0.9 (−1.4–2.5)	−0.6 (−1.5–0.1)	0.14
BMI [kg/m^2^]	0.2 (−0.7–0.9)	0.4 (−0.4–1.1)	−0.2 (−0.6–0.1)	0.17
Waist circumference [cm]	−1.0 (−4.0–1.0)	0.0 (−3.0–3.0)	−1 (−3–0)	0.025
Hip circumference [cm]	0.0 (−3.0–1.0)	0.0 (−2.0–2.0)	−1 (−2–0)	0.047
WHR	0.00 (−0.02–0.02)	0.00 (−0.02–0.02)	0.00 (−0.01–0.01)	0.81
Total body				
FM [g]	177 (−935–1,442)	862 (−975–2,292)	−523 (−1,198–197)	0.17
FM [%]	0.4 (−1.1–1.9)	1.0 (−0.9–2.4)	−0.5 (−1.1–0.2)	0.18
VAT				
Mass [g]	−14 (−68–52)	3 (−53–80)	−19 (−49–13)	0.24
Volume [cm^3^]	−15 (−77–56)	3 (−57–86)	−23 (−58–11)	0.17
Area [cm^2^]	−3.0 (−14.0–10.9)	2.0 (−10.0–18.0)	−5.0 (−11.8–1.1)	0.12
LMI [kg/m^2^]	0.0 (−0.6–0.5)	0.0 (−0.3–0.4)	−0.1 (−0.3–0.1)	0.50
ALMI [kg/m^2^]	−0.10 (−0.36–0.10)	−0.13 (−0.28–0.06)	−0.02 (−0.11–0.08)	0.76

ALMI, appendicular lean mass index; BMI, body mass index; CI, confidence interval; FM, fat mass; LMI, lean mass index; Q1–Q3, interquartile range; VAT, visceral adipose tissue; WHR, waist-to-hip ratio.

**Table 8:** Comparison of changes (Δ; post-intervention minus pre-intervention) in blood pressure and biochemical parameters between the intervention and control groups.

Variables	Intervention group (*n* = 93), median (Q1–Q3)	Control group (*n* = 94)^a^, median (Q1–Q3)	Median differences (95%CI)	*p*
Blood pressure				
SBP [mmHg]	−2 (−12–7)	−4 (−11–7)	0 (−4–5)	0.94
DBP [mmHg]	0 (−5–7)	−1 (−6–5)	1 (−2–4)	0.48
HR [bpm]	−3 (−9–2)	−3 (−10–3)	0 (−4–3)	0.94
Glucose and insulin homeostasis				
Glucose [mg/dl]	−1 (−7–5)	−1 (−5–4)	0 (−3–2)	0.79
Insulin [μIU/ml]	−0.1 (−1.1–1.6)	−0.4 (−2.0–1.5)	0.4 (−0.4–1.2)	0.31
Lipid profile				
TC [mg/dl]	−2 (−16–16)	−3 (−23–12)	4 (−4–13)	0.31
HDL-C [mg/dl]	2 (−3–5)	2 (−4–5)	0 (−2–2)	0.97
non-HDL-C [mg/dl]	−2 (−18–15)	−2 (−18–8)	3 (−4–12)	0.33
LDL-C [mg/dl]	−3 (−13–13)	−2 (−20–8)	4 (−3–12)	0.24
TG [mg/dl]	−2 (−20–27)	5 (−12–26)	−4 (−15–6)	0.42
Inflammatory parameters				
CRP [mg/l]	−0.1 (−0.6–0.1)	−0.1 (−0.3–0.3)	−0.1 (−0.3–0.1)	0.26
IL-6 [pg/ml]	0.0 (0.0–0.3)	0.0 (−0.3–0.4)	0.0 (−0.2–0.1)	0.60
IL1R1 [pg/ml]	−79.8 (−177.7–27.4)	−52.9 (−151.9–11.1)	−15.1 (−61.3–32.3)	0.51
TNF-α [pg/ml]	0.3 (−0.6–0.9)	0.0 (−0.5–1.2)	0.0 (−0.4–0.4)	0.87
Peripheral neurodegeneration parameters				
BDNF [ng/ml]	−0.3 (−3.7–1.9)	−0.7 (−3.7–1.9)	0.1 (−1.2–1.3)	0.94
Tau protein [pg/ml]	1.1 (−6.1–8.7)	0.7 (−9.2–7.4)	0.9 (−2.8–4.7)	0.62
β-amyloid 1–40 [pg/ml]	30.4 (−5.9–78.4)	36.2 (−0.7–79.9)	−7.2 (−25.8–10.0)	0.42
β-amyloid 1–42 [pg/ml]	0.6 (−0.4–2.0)	0.3 (−0.6–2.3)	0.1 (−0.5–0.7)	0.63
β-amyloid 1–42/1–40	0.00 (−0.01–0.01)	0.00 (−0.01–0.00)	0.00 (0.00 – −0.01)	0.55

BDNF, brain-derived neurotrophic factor; CI, confidence interval; CRP, C reactive protein; DBP, diastolic blood pressure; HDL-C, high density lipoprotein cholesterol; HR, heart rate; IL, interleukin; LDL-C, low density lipoprotein cholesterol; non-HDL-C, total cholesterol minus high density lipoprotein cholesterol; Q1 – Q3, interquartile range; SBP, systolic blood pressure; TC, total cholesterol; TG, triglycerides; TNF-α, tumour necrosis factor α.

a One subject refused blood sampling.

## 4 Discussion

In the present study, we showed significant between-group differences in changes in physical activity levels (median difference in daily step count [95%CI]: 1,728 [1,207 to 2,271]) over the 12-month intervention. Although these differences were statistically significant, their clinical relevance requires confirmation in future studies, as we observed only modest effects on cognitive performance and anthropometric parameters in individuals with MCI.

In our study, the group that increased its daily step count (median [Q1 – Q3]: 1,474 [207–3,313]) showed changes in cognitive function, with significant between-group differences in changes in MoCA test scores and SWM test outcomes. These results are partly consistent with previous studies. The effects of physical activity and exercise on cognitive function in individuals with MCI were summarised in a recent umbrella review by Demurtas et al. (2020). Evidence from both observational and intervention studies suggests that physical activity improves cognitive function, although the magnitude of the benefits varies by the type of activity performed. Positive effects were particularly noted in the domains of attention, executive function, and memory. A systematic review of randomised controlled trials also suggested that increased physical activity may improve global cognition, executive functioning, attention, and memory in individuals with MCI but has no effect in individuals with dementia (Öhman et al., 2014). In contrast, a recent meta-analysis of randomised controlled trials conducted by Lin et al. (2023) did not detect any benefit of walking for improving cognitive function in individuals with MCI. However, this may be attributable to heterogeneity in diagnostic criteria, MCI types, and intervention characteristics across the included studies. Despite numerous studies confirming the beneficial effects of physical activity on cognitive function, there is a lack of randomised controlled trials with long-term intervention periods that assess the impact of walking on cognitive outcomes. The current study makes an important contribution to assessing the impact of physical activity on MCI. Our findings suggest that even a small increase in daily activity (median difference [95%CI]: 1,728 [1,207 to 2,271] steps/day) may affect cognitive function, although the observed effect appears modest. Therefore, the clinical significance of these findings requires confirmation in future studies and should be interpreted with caution, particularly given measurement variability and the minimal detectable change. Changes in lifestyle factors, such as diet, medication use, sleep, smoking, alcohol consumption, stress, and others, may have influenced our results. In addition, repeated cognitive testing may be associated with practice effects, which may also influence the findings.

Previous studies suggested that physical activity may be positively associated with brain volume, with the hippocampus being more sensitive to the effects of exercise (Erickson et al., 2014). In a meta-analysis of controlled trials conducted by Wilckens et al. (2021), the authors found that moderate physical activity interventions lasting longer than six months may affect hippocampal volume, particularly in older adults (≥65 years). Another meta-analysis found that physical activity interventions may attenuate hippocampal atrophy only in individuals with MCI but not in individuals with Alzheimer’s disease (Kress et al., 2024). Varma et al. (2015) found that the amount, duration, and frequency of low-intensity daily walking may be associated with hippocampal volume, but only in women. In contrast to these findings, our results did not support the hypothesis that increasing daily step count affects brain volume. However, it is worth noting that total hippocampus volume showed a trend towards changes (*p* = 0.087). The moderate baseline physical activity among the study participants may partially explain these results.

The present trial did not observe any effect of the intervention on neurodegenerative biomarkers, such as BDNF, tau protein, amyloid β-40 and amyloid β-42. However, in our study, these parameters were measured in peripheral blood rather than in cerebrospinal fluid, the standard specimen for assessing cognitive disorders. Therefore, the effect of physical activity on neurodegenerative markers warrants further investigation, as previous studies have reported inconsistent findings (Szuhany et al., 2015; Rodriguez-Ayllon et al., 2024).

The beneficial effect of walking on anthropometric indices and body composition has been previously reported. A meta-analysis of randomised controlled trials and cohort studies conducted by Richardson et al. (2008) included studies of individuals with excessive body weight and intervention periods longer than 4 weeks, also reported weight loss associated with walking interventions, with an average body weight reduction of 0.05 kg per week, an estimated mean weight change of −1.27 kg, and a greater effect observed in studies of longer duration. Notably, participants exceeded 10,000 steps per day in only two of the nine studies included in this review. The present study revealed changes in waist and hip circumferences, but there were no significant between-group differences in other anthropometric and body composition parameters. However, these results should be interpreted with caution given the exploratory nature of these analyses.

In this study, a small increase in step count did not affect glucose, insulin, lipid metabolism, or blood pressure. A recent meta-analysis of observational and intervention studies by Bravata et al. (2007) also suggested that walking does not significantly improve the lipid profile or fasting glucose levels, although a positive effect was observed on SBP.

Similarly, no effects of our intervention on inflammatory markers were observed in this trial, despite previous studies reporting beneficial effects (Ma et al., 2022). The relatively high baseline physical activity among study participants may partly explain the lack of an effect of physical activity on inflammatory markers observed in the present study. Low adherence to the study intervention may have influenced our findings.

The lack of an effect of our intervention on brain volume, biochemical parameters, and body composition may be associated with the low physiological stimulus it provided, as no specific recommendations regarding its intensity were provided. Therefore, it is possible that the achieved increase in physical activity was insufficient to induce detectable physiological or neurobiological adaptations. In addition, the moderate baseline physical activity among the study participants may affect our findings.

This study has some limitations and strengths. First, MCI was identified solely from MoCA test scores, with participants grouped based on a single cognitive function assessment, without comparison with peers of similar sex, age, or education level. The participants did not undergo a formal neurological, neuropsychological, or functional assessment either. Other limitations include the brief baseline measurement period for physical activity before the intervention, along with relatively high baseline physical activity. Also, the limited validity of physical activity data assessed by the Fitbit Inspire HR tracker is an important study limitation. On the other hand, a recent study assessed the validity of the Fitbit Inspire HR and found good agreement between Fitbit-derived step counts and total physical activity and the ActiGraph GT3X. However, the agreement for moderate-to-vigorous physical activity was weaker, suggesting that estimates of activity intensity should be interpreted with caution (Polhemus et al., 2023). In addition, a significant correlation was observed between energy expenditure estimates from the Fitbit Inspire HR and metabolic measurements (Van Camp, 2020). Additionally, due to differences in smartphone models and operating systems, it was not possible to ensure that all participants used the same version of the Fitbit application throughout the study. Another limitation is that neurodegeneration markers were measured in blood rather than cerebrospinal fluid. Moreover, the inclusion of Caucasian, community-dwelling participants aged 50–70 years with MCI who owned a smartphone may be a potential source of bias and may limit the generalisability of the obtained findings. Furthermore, although minor between-group differences were observed at baseline, analyses limited to participants who completed the trial indicated comparable group characteristics. Importantly, although we planned to assess sleep data, these data were ultimately not analysed due to difficulties in data collection and a substantial proportion of missing data. Nevertheless, sleep disturbances may influence cognitive performance and may therefore represent a relevant confounding factor in our study (Künstler et al., 2023). We also did not assess changes in medication use and depressive symptoms during the intervention period, although these factors could have affected our results. Moreover, we did not collect data on severe acute respiratory syndrome coronavirus 2 (SARS-CoV-2) infections. However, severe infection may be associated with subsequent cognitive impairment (Magnúsdóttir et al., 2026). Therefore, differences in SARS-CoV-2 exposure between the groups may have influenced our findings. Moreover, although the Wilcoxon signed-rank test was additionally performed to assess within-group changes over time, this analysis has limitations, as statistically significant changes within a group do not necessarily indicate a difference in the magnitude of between-group changes. Therefore, our primary analysis was based on between-group comparisons of changes, assessed using the Mann–Whitney test. Finally, because several secondary outcomes were evaluated and no adjustment for multiple comparisons was implemented, the risk of type I error and false-positive findings is increased. Therefore, the analyses of secondary outcomes should be considered exploratory, and statistically significant findings should not be interpreted as confirmatory evidence.

The study was a single-centre, single-blind, randomised, controlled, parallel-group trial, with participants recruited through newsletters, websites, posters, and email invitations sent to companies, offices, and institutions to reach their employees. Physical activity was objectively and continuously monitored throughout the entire 12-month intervention period in a large, well-characterised, and homogenous MCI population. The study created a unique opportunity to assess subjects with moderate, rather than low, activity levels, characterised by minor changes in daily step count, physical activity, and energy expenditure. The study findings are partly generalisable to similar populations. However, the study’s strict inclusion and exclusion criteria, along with the relatively homogenous sample, enhance internal validity but may limit generalisability to populations with more diverse health profiles, as we excluded individuals with significant comorbidities or those taking cognitive-enhancing medications. The predominance of female participants may limit the applicability of the results to a more sex-balanced or male-dominant population.

In conclusion, an increase in daily step counts during the 12-month intervention period affected cognitive function and altered anthropometric parameters in Caucasian, community-dwelling individuals aged 50–70 years with MCI who owned a smartphone. However, the clinical relevance of these changes requires confirmation in future studies. Notably, these effects were observed in a population with moderate baseline activity levels and only minor increases in daily step count, physical activity, and energy expenditure. In contrast, such modest changes in daily physical activity did not affect brain volume, markers of neurodegeneration, glucose, insulin, and lipid metabolism, inflammation, blood pressure, or body composition. However, the intensity of our intervention may not have been sufficient to induce detectable physiological or neurobiological changes.

## Data Availability

The datasets presented in this article are not readily available because participants did not consent to data sharing. Requests to access the datasets should be directed to JW, jarwalk@ump.edu.pl.

## Supplementary material

SUPPLEMENTARY FIGURE 1 Daily step count over the 12-month intervention period in the intervention and control groups. Data are presented as median and interquartile range.

SUPPLEMENTARY FIGURE 2 Daily sedentary behaviour over the 12-month intervention period in the intervention and control groups. Data are presented as median and interquartile range.

SUPPLEMENTARY FIGURE 3 Daily light activity over the 12-month intervention period in the intervention and control groups. Data are presented as median and interquartile range.

SUPPLEMENTARY FIGURE 4 Daily moderate activity over the 12-month intervention period in the intervention and control groups. Data are presented as median and interquartile range.

SUPPLEMENTARY FIGURE 5 Daily vigorous activity over the 12-month intervention period in the intervention and control groups. Data are presented as median and interquartile range.

SUPPLEMENTARY FIGURE 6 Scatter plots showing the relationship between average sedentary behaviour and moderate-to-vigorous physical activity over the 12-month intervention period. **(A)** Control group (Spearman's *rho* = -0.13, *p* = 0.20). **(B)** Intervention group (Spearman's *rho* = -0.18, *p* = 0.079).

## Glossary

ALMI: appendicular lean mass index
API: application programming interface
BDNF: brain-derived neurotrophic factor
BMI: body mass index
CANTAB: Cambridge Neuropsychological Test Automated Battery
CRP: C-reactive protein
DBP: diastolic blood pressure
DMS: Delayed Matching to Sample
ELISA: enzyme-linked immunosorbent assay
FM: fat mass
HAM-D: Hamilton Depression Rating Scale
HDL-C: high-density lipoprotein cholesterol
HR: heart rate
IL-6: interleukin-6
IL1R1: interleukin-1 receptor type α
IQR (Q1–Q3): interquartile range
LDL-C: low-density lipoprotein cholesterol
LMI: lean mass index
MCI: mild cognitive impairment
MoCA: Montreal Cognitive Assessment Test
MOT: Motor Screening Task
PAL: Paired Associates Learning
PRM: Pattern Recognition Memory
RTI: Reaction Time
RVP: Rapid Visual Information Processing
SARS-CoV-2: severe acute respiratory syndrome coronavirus 2
SBP: systolic blood pressure
SD: standard deviation
SWM: Spatial Working Memory
TC: total cholesterol
TG: triglycerides
TNFα: tumour necrosis factor α
VAT: visceral adipose tissue
WHR: waist-to-hip ratio
95%CI: 95% confidence intervals
%FM: percentage of fat mass
