# Supplementary material for: Increasing daily step count in mild cognitive impairment: a 12-month randomised controlled trial
Source: Front Aging Neurosci. 2026 Sep 23;18:1958222. doi: 10.3389/fnagi.2026.1958222 (PMC13642802; doi:10.3389/fnagi.2026.1958222)
Supplement: Supplementary file 1 [file Data_Sheet_1.DOCX]

Supplementary Material

# 1 Supplementary Methods

The MOT test evaluates whether sensorimotor deficits or a lack of comprehension may affect valid data collection. During the test, crosses appear in different locations on the screen, and the participant must select them as quickly and accurately as possible. The results are presented as the mean latency for accurate responses to the presented crosses, measured in milliseconds.

The DMS test assesses the ability to visually match patterns simultaneously and retain short-term visual memory for designs that cannot be described verbally. During the test, the participant was required to correctly select the pattern that had been previously presented to them. The sample and list of patterns are presented either simultaneously or after a delay of 0, 4, or 12 seconds, with the results presented as the percentage of correct answers on the first trial, with target and response stimuli presented either simultaneously or after delays of 0, 4, and 12 seconds. Additionally, the probability of an incorrect answer following a previous incorrect response was assessed.

The PAL test evaluates visual memory and the ability to learn and recognise new visual patterns. During the test, participants view boxes that open in a random order, with some boxes revealing a specific pattern, and the participant must choose the box where each pattern was previously shown. If a participant makes a mistake, the boxes are reopened to help them recall the locations of the patterns. The test results are presented as the number of correctly chosen boxes after the initial recall of the pattern locations, along with the number of incorrectly chosen boxes, including adjustments for potential errors that could occur in subsequent trials, attempts, and recalls that were not reached.

The PRM test evaluates visual pattern recognition memory. Participants are shown a series of visual patterns, and they must select between a previously seen pattern and a new one. A second recognition phase is conducted following a delay period, with the results reported as the number of correct patterns identified in each phase.

The RTI test measures motor and mental response speeds, as well as accuracy and impulsivity. During the test, the participant holds down a button at the bottom of the screen until a yellow dot appears in one of the circles above the button. The participant must then release the button and touch the circle where the dot appeared, with outcomes reported as the median reaction time and movement time.

In the RVP test, participants must detect a target sequence of digits to assess sustained attention. The results are expressed as a signal called A’ which measure the participant's sensitivity to detect the target sequences), the probability of false alarms, and the median latency of correct responses.

The goal of the SWM test is to evaluate participants' spatial working memory by requiring them to find a yellow token hidden in one of the squares presented on the screen. The test evaluates participants' search strategies by measuring the number of times the participants initiated a new search pattern from the same square from which they had previously started. Additionally, the test assesses how often the participant revisits a square where a token had already been found (Cambridge Cognition, 2024).

References:

1. Cambridge Cognition. (2024). Digital cognitive assessments. https://cambridgecognition.com/digital-cognitive-assessments/ (Accessed November 10, 2024).

# 2 Supplementary Tables

**Supplementary Table 1. CONSORT 2025 checklist.**

| Section / Topic | No | CONSORT 2025 checklist item description | Reported on page no. |
| --- | --- | --- | --- |
| Title and abstract | | |  |
| Title and structured abstract | 1a | Identification as a randomized trial | 1, 2 |
|  | 1b | Structured summary of the trial design, methods, results, and conclusions | 2 |
| Open science | | |  |
| Trial registration | 2 | Name of trial registry, identifying number (with URL) and date of registration | 2, 3 |
| Protocol and statistical analysis plan | 3 | Where the trial protocol and statistical analysis plan can be accessed | 3 |
| Data sharing | 4 | Where and how the individual de-identified participant data (including data dictionary), statistical code and any other materials can be accessed | 16 |
| Funding and conflicts of interest | 5a | Sources of funding and other support (e.g., supply of drugs), and role of funders in the design, conduct, analysis and reporting of the trial | 2, 12 |
|  | 5b | Financial and other conflicts of interest of the manuscript authors | 12 |
| Introduction | | |  |
| Background and rationale | 6 | Scientific background and rationale | 2-3 |
| Objectives | 7 | Specific objectives related to benefits and harms | 3 |
| Methods | | |  |
| Patient and public involvement | 8 | Details of patient or public involvement in the design, conduct and reporting of the trial | 3 |
| Trial design | 9 | Description of trial design including type of trial (e.g., parallel group, crossover), allocation ratio, and framework (e.g., superiority, equivalence, non-inferiority, exploratory) | 3 |
| Changes to trial protocol | 10 | Important changes to the trial after it commenced including any outcomes or analyses that were not prespecified, with reason | - |
| Trial setting | 11 | Settings (e.g., community, hospital) and locations (e.g., countries, sites) where the trial was conducted | 3 |
| Eligibility criteria | 12a | Eligibility criteria for participants | 4 |
|  | 12b | If applicable, eligibility criteria for sites and for individuals delivering the interventions (e.g., surgeons, physiotherapists) | - |
| Intervention and comparator | 13 | Intervention and comparator with sufficient details to allow replication. If relevant, where additional materials describing the intervention and comparator (e.g., intervention manual) can be accessed | 4-5 |
| Outcomes | 14 | Pre-specified primary and secondary outcomes, including the specific measurement variable (e.g., systolic blood pressure), analysis metric (e.g., change from baseline, final value, time to event), method of aggregation (e.g., median, proportion), and time point for each outcome | 5-8 |
| Harms | 15 | How harms were defined and assessed (e.g., systematically, non-systematically) | 5 |
| Sample size | 16a | How sample size was determined, including all assumptions supporting the sample size calculation | 8 |
|  | 16b | Explanation of any interim analyses and stopping guidelines | - |
| Randomization: |  |  |  |
| Sequence generation | 17a | Who generated the random allocation sequence and the method used | 4 |
|  | 17b | Type of randomization and details of any restriction (e.g., stratification, blocking and block size) | 4 |
| Allocation concealment mechanism | 18 | Mechanism used to implement the random allocation sequence (e.g., central computer/telephone; sequentially numbered, opaque, sealed containers), describing any steps to conceal the sequence until interventions were assigned | 4 |
| Implementation | 19 | Whether the personnel who enrolled and those who assigned participants to the interventions had access to the random allocation sequence | 4 |
| Blinding | 20a | Who was blinded after assignment to interventions (e.g., participants, care providers, outcome assessors, data analysts) | 4 |
|  | 20b | If blinded, how blinding was achieved and description of the similarity of interventions | 4 |
| Statistical methods | 21a | Statistical methods used to compare groups for primary and secondary outcomes, including harms | 8 |
|  | 21b | Definition of who is included in each analysis (e.g., all randomized participants), and in which group | 8 |
|  | 21c | How missing data were handled in the analysis | 8 |
|  | 21d | Methods for any additional analyses (e.g., subgroup and sensitivity analyses), distinguishing prespecified from post-hoc | 8 |
| Results | | |  |
| Participant flow, including flow diagram | 22a | For each group, the numbers of participants who were randomly assigned, received intended intervention, and were analyzed for the primary outcome | Figure 1 |
|  | 22b | For each group, losses and exclusions after randomization, together with reasons | Figure 1 |
| Recruitment | 23a | Dates defining the periods of recruitment and follow-up for outcomes of benefits and harms | 8 |
|  | 23b | If relevant, why the trial ended or was stopped | - |
| Intervention and comparator delivery | 24a | Intervention and comparator as they were actually administered (e.g., where appropriate, who delivered the intervention/comparator, how participants adhered, whether they were delivered as intended) | 3-5 |
|  | 24b | Concomitant care received during the trial for each group | - |
| Baseline data | 25 | A table showing baseline demographic and clinical characteristics for each group | Tables 1-2 |
| Numbers analysed,  outcomes and estimation | 26 | For each primary and secondary outcome, by group:   - the number of participants included in the analysis - the number of participants with available data at the outcome time point - result for each group, and the estimated effect size and its precision (such as 95% confidence interval) - for binary outcomes, presentation of both absolute and relative effect size | Tables 3-7 |
| Harms | 27 | All harms or unintended events in each group | 18 |
| Ancillary analyses | 28 | Any other analyses performed, including subgroup and sensitivity analyses, distinguishing pre-specified from post-hoc | Supplementary Table 2-3 |
| Discussion | | |  |
| Interpretation | 29 | Interpretation consistent with results, balancing benefits and harms, and considering other relevant evidence | 9-12 |
| Limitations | 30 | Trial limitations, addressing sources of potential bias, imprecision, generalizability, and, if relevant, multiplicity of analyses | 11 |

Note: Reproduced from Hopewell et al. (2025). Licensed under Creative Commons Attribution 4.0 (CC BY 4.0).

**Supplementary Table 2. Intervention effect on cognitive test results.**

|  | **Intervention group (n = 92)^a^, median (Q1 – Q3)** | | **p** | **Control group (n = 95), median (Q1 – Q3)** | | **p** |
| --- | --- | --- | --- | --- | --- | --- |
|  | **Pre-intervention** | **Post-intervention** |  | **Pre-intervention** | **Post-intervention** |  |
| MoCA score [points]^b^ | 25 (23 – 26) | 26 (24 – 27) | <0.0001 | 25 (24 – 26) | 25 (23 – 26) | 0.44 |
| DMS | | | | | | |
| Percent correct, simultaneous | 100 (100 – 100) | 100 (100 – 100) | 0.8689 | 100 (100 – 100) | 100 (100 – 100) | 10.0 |
| Percent correct, 0-s delay | 100 (80 – 100) | 100 (80 – 100) | 0.9477 | 100 (80 – 100) | 100 (80 – 100) | 0.31 |
| Percent correct, 4-s delay | 80 (60 – 100) | 100 (80 – 100) | 0.0212 | 80 (80 – 100) | 80 (80 – 100) | 0.47 |
| Percent correct, 12-s delay | 80 (60 – 100) | 80 (80 – 100) | 0.3467 | 80 (60 – 100) | 80 (60 – 100) | 0.89 |
| Probability of error given error | 0.0 (0.0 – 0.2) | 0.0 (0.0 – 0.0) | 0.6664 | 0.0 (0.0 – 0.0) | 0.0 (0.0 – 0.2) | 0.060 |
| MOT | | | | | | |
| Mean latency [ms] | 793.8 (731.1 – 953.6) | 776.8 (698.7 – 953.0) | 0.4025 | 815.5 (732.4 – 981.6) | 782.3 (665.2 – 909.2) | 0.028 |
| PAL | | | | | | |
| Total errors, adjusted | 20 (10 – 31) | 15 (9 – 21) | 0.0003 | 21 (13 – 37) | 17 (11 – 25) | <0.0001 |
| First-attempt memory score | 10 (8 – 12) | 12 (9 – 14) | <0.0001 | 10 (7 – 13) | 11 (9 – 14) | 0.0017 |
| PRM | | | | | | |
| Percent correct immediate | 88.9 (77.8 – 94.4) | 88.9 (83.3 – 100.0) | 0.0204 | 88.9 (77.8 – 94.4) | 88.9 (83.3 – 94.4) | 0.37 |
| Percent correct delayed | 86.1 (77.8 – 94.4) | 88.9 (77.8 – 94.4) | 0.8591 | 83.3 (72.2 – 88.9) | 83.33 (72.2 – 94.4) | 0.41 |
| RTI | | | | | | |
| Median five-choice reaction time [ms] | 395.0 (368.3 – 426.0) | 393.0 (365.3 – 427.8) | 0.6689 | 395.0 (363.0 – 423.0) | 389.5 (362.0 – 410.5) | 0.16 |
| Median five-choice movement time [ms] | 303.8 (257.5 – 360.8) | 310.0 (252.0 – 365.8) | 0.5489 | 292.5 (253.5 – 352.0) | 312.5 (268.5 – 359.5) | 0.044 |
| RVP | | | | | | |
| A’ | 0.8772 (0.8438 – 0.9120) | 0.8969 (0.8647 – 0.9313) | 0.0001 | 0.8716 (0.8405 – 0.9006) | 0.8837 (0.8559 – 0.9092) | 0.037 |
| False alarm probability | 0.0060 (0.0023 – 0.0108) | 0.0050 (0.0019 – 0.0131) | 0.5875 | 0.0064 (0.0021 – 0.0251) | 0.0057 (0.0020 – 0.0162) | 0.0046 |
| Median response latency [ms] | 499.0 (458.0 – 573.5) | 487.2 (438.0 – 563.0) | 0.1688 | 537.8 (465.0 – 599.5) | 515.0 (449.5 – 615.0) | 0.27 |
| SWM | | | | | | |
| Strategy | 10 (8 – 11) | 9 (7 – 10) | 0.0018 | 10 (9 – 11) | 9 (8 – 11) | 0.25 |
| Between errors, 4 boxes | 1 (0 – 2) | 0 (0 – 1) | 0.0035 | 1 (0 – 2) | 0 (0 – 2) | 0.35 |
| Between errors, 6 boxes | 6 (2 – 8) | 4 (0 – 7) | 0.0023 | 5 (2 – 7) | 5 (2 – 7) | 0.59 |
| Between errors, 8 boxes | 12 (7 – 15) | 10 (4 – 14) | 0.0270 | 14 (8 – 15) | 13 (10 – 15) | 0.87 |

DMS, delayed match to sample; MoCA, Montreal Cognitive Assessment Scale; MOT, motor screening task; PAL, paired associates learning; PRM, pattern recognition memory; Q1 – Q3, interquartile range; RTI, reaction time task; RVP, rapid visual information processing; SWM, spatial working memory

^a^One participant declined to complete the Cambridge Neuropsychological Test Automated Battery (CANTAB) examination at the end of the intervention (*n* = 92); ^b^*n* = 93 (intervention group)

**Supplementary Table 3. Intervention effect on brain quantitative structural measures derived from magnetic resonance imaging.**

|  | **Intervention group (n = 93, median (Q1 – Q3)** | | ***p*** | **Control group (n = 94)**^a^, **median (Q1 – Q3)** | | ***p*** |
| --- | --- | --- | --- | --- | --- | --- |
|  | **Pre-intervention** | **Post-intervention** |  | **Pre-intervention** | **Post-intervention** |  |
| Cortex volume [mm^3^] | 449,268 (432,801 – 482,997) | 449,161 (427,523 – 483,829) | 0.0008 | 449,962 (426,603 – 474,957) | 447,737 (421,472 – 471,058) | 0.0008 |
| Total grey matter volume [mm^3^] | 613,481 (589,398 – 649,706) | 609,005 (585,769 – 650,773) | 0.0001 | 610,969 (582,363 – 642,615) | 605,897 (578,584 – 643,243) | <0.0001 |
| Subcortical grey matter volume [mm^3^] | 58,585 (55821 – 62,408) | 58,478 (55,642 – 62,138) | 0.0003 | 58,441 (55,847 – 62,120) | 58,071 (55,500 – 61,605) | <0.0001 |
| Total lateral ventricle [mm^3^]^b^ | 8782 (6207 – 12,199) | 8600 (6330 – 11,781) | <0.0001 | 8158 (6563 – 11,723) | 8546 (6848 – 12,109) | <0.0001 |
| Total hippocampus [mm^3^]^b^ | 4230 (3997 – 4545) | 4249 (3989 – 4568) | 0.2297 | 4232 (3951 – 4588) | 4195 (3907 – 4491) | 0.0004 |

Q1 – Q3, interquartile range

^a^One participant refused to undergo the examination at the end of the intervention; ^b^(Left+right)/2
